# Cadmium Induces Vascular Endothelial Cell Detachment by Downregulating Claudin-5 and ZO-1 Levels

**DOI:** 10.3390/ijms252011035

**Published:** 2024-10-14

**Authors:** Takato Hara, Mayuka Asatsu, Tatsuya Yamagishi, Chinami Ohata, Hitomi Funatsu, Yuzuki Takahashi, Misaki Shirai, Chiaki Nakata, Haruka Katayama, Toshiyuki Kaji, Tomoya Fujie, Chika Yamamoto

**Affiliations:** 1 Faculty of Pharmaceutical Sciences, Toho University, 2-2-1 Miyama, Funabashi 274-8510, Chiba, Japan3023004s@st.toho-u.ac.jp (M.S.);; 2 Faculty of Pharmaceutical Sciences, Tokyo University of Sciences, 2641 Yamazaki, Noda 278-8510, Chiba, Japan; t-kaji@rs.tus.ac.jp (T.K.); t-fujie@rs.tus.ac.jp (T.F.)

**Keywords:** cadmium, vascular endothelial cell, tight junction, claudin-5, ZO-1

## Abstract

Cadmium is a contributing factor to cardiovascular diseases and highly toxic to vascular endothelial cells. It has a distinct mode of injury, causing the de-endothelialization of regions in the monolayer structure of endothelial cells in a concentration-dependent manner. However, the specific molecules involved in the cadmium toxicity of endothelial cells remain unclear. The purpose of this study was to identify the specific molecular mechanisms through which cadmium affects endothelial detachment. Cadmium inhibited the expression of claudin-5 and zonula occludens (ZO)-1, which are components of tight junctions (strongest contributors to intercellular adhesion), in a concentration- and time-dependent manner. Compared to arsenite, zinc, and manganese, only cadmium suppressed the expression of both claudin-5 and ZO-1 molecules. Moreover, the knockdown of claudin-5 and ZO-1 exacerbated cadmium-induced endothelial cell injury and expansion of the detachment area, whereas their overexpression reversed these effects. CRE-binding protein inhibition reduced cadmium toxicity, suggesting that CRE-binding protein activation is involved in the cadmium-induced inhibition of claudin-5 and ZO-1 expression and endothelial detachment. These findings provide new insights into the toxicological mechanisms of cadmium-induced endothelial injury and risk of cardiovascular disease.

## 1. Introduction

Cadmium is an environmental pollutant. Humans are exposed to it via diet and smoking, agricultural products, especially rice and tobacco, and via absorbing it during growth. As cadmium is mainly accumulated in the liver and kidneys, causing significant histopathological damage, many research groups have investigated these organs [1,2]. However, it is necessary for cadmium to pass through blood vessels to reach these organs. When endothelial cells are damaged and subendothelial tissue is exposed, the risk of thrombosis and cardiovascular disease due to platelet aggregation increases [3,4]. Cadmium induces atherosclerotic lesions with lipid plaque formation [5]. Epidemiological studies have reported that cadmium is a contributing factor to atherosclerosis [6,7]. Using cultured vascular endothelial cells, we previously reported that (1) exposure to cadmium causes circular detachment from monolayered endothelial cells and (2) injury involving cell detachment is cadmium-specific and not observed with other heavy metals [8,9,10]. However, the detailed mechanisms underlying the induction of such injuries remain unknown.

Intercellular junctions include many types, mainly tight junctions, adherens junctions, desmosomes, and gap junctions. Among these types, tight junctions contribute the most to cell–cell adhesion, sealing the gaps on the apical side between adjacent cells and restricting the passage of substances [11]. Claudins are transmembrane proteins that associate with other proteins, such as zippers, and form tight junctions. Other transmembrane proteins include occludin and JAMs, and the backbone molecule, zonula occludens, is responsible for linking claudins and occludin to the cytoskeletal actin [12,13,14]. We hypothesized that monolayers of cultured vascular endothelial cells exposed to cadmium would form circular detachments owing to the weakening of tight junctions. To verify this, we analyzed the changes in the expression levels of tight junction components in vascular endothelial cells exposed to cadmium and the effects of the expression levels of these molecules on endothelial cell detachment induced by cadmium.

## 2. Results

### 2.1. Cadmium Inhibits Claudin-5 and ZO-1 Expression in Vascular Endothelial Cells

**Figure 1:**
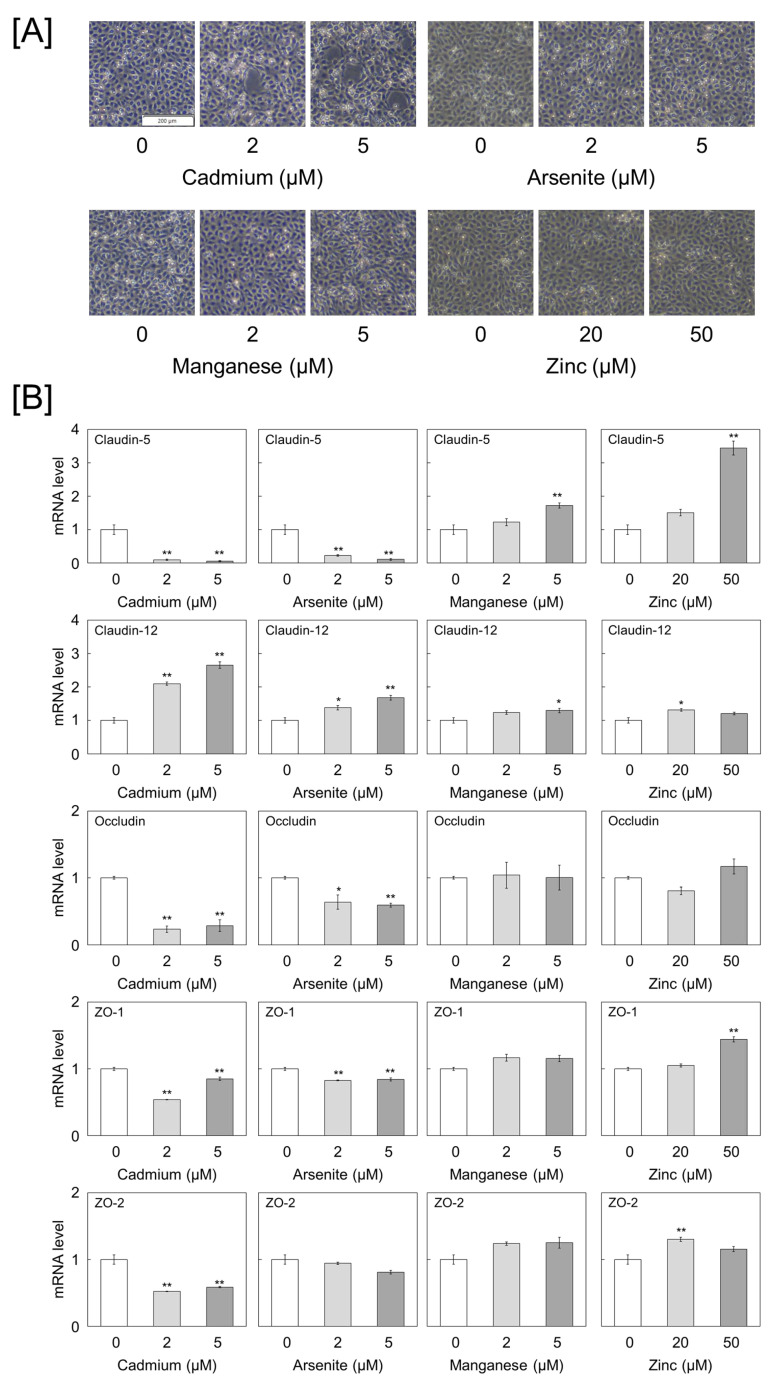
Effects of metals on the morphology and mRNA expression levels of tight junction molecules in vascular endothelial cells. (**A**) Morphology of vascular endothelial cells. (**B**) mRNA expression levels of claudin-5, claudin-12, occludin, zonula occludens (ZO)-1, and ZO-2 in cells treated with 2 and 5 μM cadmium, arsenite, and manganase or 20 and 50 μM zinc for 24 h. Values are represented as the mean ± standard error (S.E.) of triplicates. * *p* < 0.05 and ** *p* < 0.01 vs. control by Dunnett’s test.

As a preliminary step, mRNA expression levels of tight junction components, namely, claudins, occludin, and ZOs, were analyzed in bovine aortic endothelial cells, which easily exhibit cellular detachment in response to cadmium. Claudin-4, -5, -7, -8, -12, -20, and -24, occludin, and ZO -1 and -2 were expressed in the cells (Supplemental Figure S1 and Supplemental Table S1). The protein expression of claudin-5 and -12, occludin, and ZO-1 and -2 was also detected. The mRNA expression levels of claudin-5 were higher than those of claudin-12, and the mRNA expression levels of ZO-1 were higher than those of ZO-2. Notably, the mRNA expression of ZO-3 was often below the detection limit, but its protein expression was detected. Subsequently, the effects of cadmium on these molecules were examined. Additionally, the effects of arsenite (toxic heavy metal) and manganese and zinc (essential trace elements with low toxicity to endothelial cells) were also examined for comparison. The area of circular detachment of endothelial cells from the monolayer increased with increasing concentrations of cadmium, consistent with a previous report [8]. However, no significant morphological changes were observed in the groups treated with other metals (Figure 1A and Supplemental Figure S2A).

**Figure 2:**
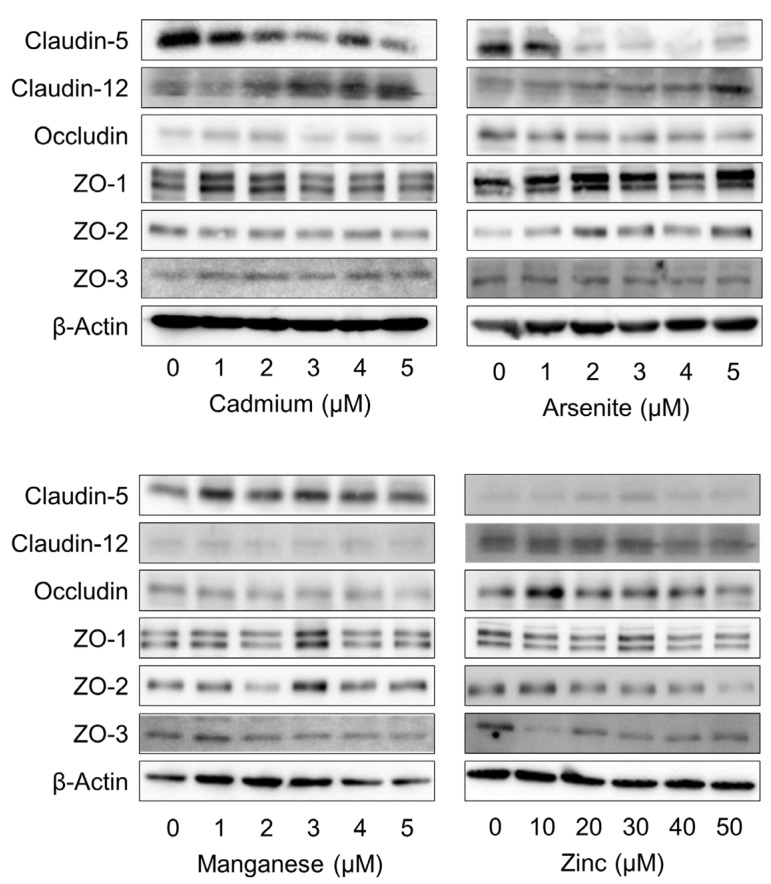
Effects of metals on the protein expression levels of tight junction molecules in vascular endothelial cells. Protein expression levels of claudin-5, claudin-12, occludin, ZO-1 (both bands are ZO-1), ZO-2, and ZO-3 in vascular endothelial cells treated with 1, 2, 3, 4, and 5 μM cadmium, arsenite, and manganase or 10, 20, 30, 40, and 50 μM of zinc for 24 h.

**Figure 3:**
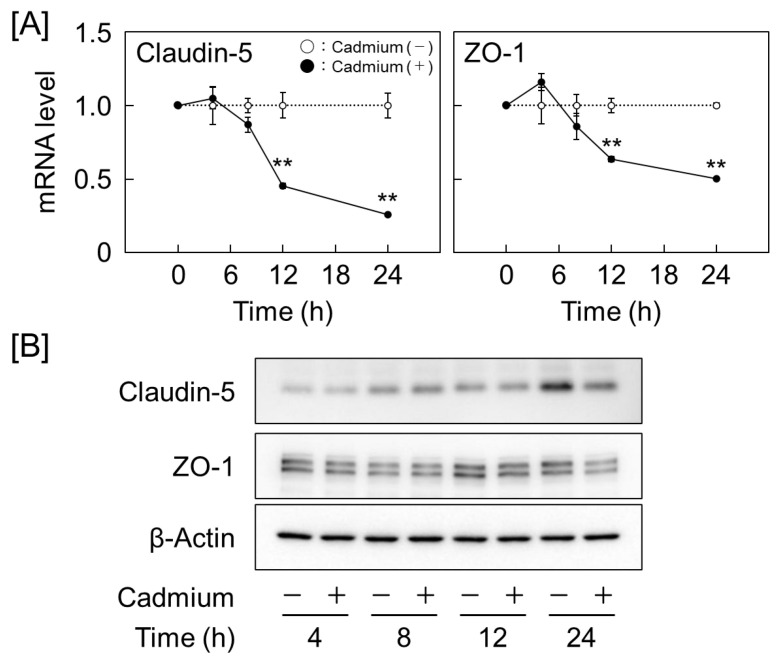
Cadmium time-dependently inhibits claudin-5 and ZO-1 expression in vascular endothelial cells. (**A**) mRNA and (**B**) protein expression levels of claudin-5 and ZO-1 in cells treated with 2 μM cadmium for 4, 8, 12, and 24 h. Values are represented as the mean ± S.E. of triplicates. ** *p* < 0.01 vs. corresponding control by Student’s *t*-test.

Next, the effects of cadmium on the mRNA expression levels of claudin-5 and -12, occludin, and ZO-1 and -2 in vascular endothelial cells were examined. Cadmium inhibited the expression of claudin-5, occludin, ZO-1, and ZO-2 but induced that of claudin-12 in a concentration-dependent manner. Although the same tendency was observed in the arsenite-treated group, the degree of inhibition and induction of each gene was weaker than that in the cadmium-treated group. In contrast, manganese and zinc did not downregulate the expression of any mRNA, and zinc increased the mRNA expression levels of claudin-5 and ZO-1 in a concentration-dependent manner (Figure 1B). Subsequently, the effects of each metal on the expression levels of these proteins were examined. Cadmium exerted similar effects on mRNA and protein expression, significantly decreasing the claudin-5, ZO-1, and ZO-2 levels and increasing the claudin-12 levels. Arsenite decreased the claudin-5 protein and mRNA expression levels and increased the ZO-1 and ZO-2 protein, but not mRNA, expression levels. Manganese and zinc increased the claudin-5 levels and decreased the ZO-3 levels. Zinc also decreased the ZO-2 level, but manganese did not (Figure 2 and Supplemental Figure S3A). As the claudin-5 and ZO-1 levels were significantly decreased by cadmium, we examined the time-dependent effects of cadmium on their expression levels. Cadmium significantly reduced the mRNA expression levels of claudin-5 and ZO-1 after 12 h of treatment and the protein expression levels at 24 h. (Figure 3 and Supplemental Figure S3B).

### 2.2. Claudin-5 and ZO-1 Are Key Molecules for Cadmium Toxicity in Vascular Endothelial Cells

**Figure 4:**
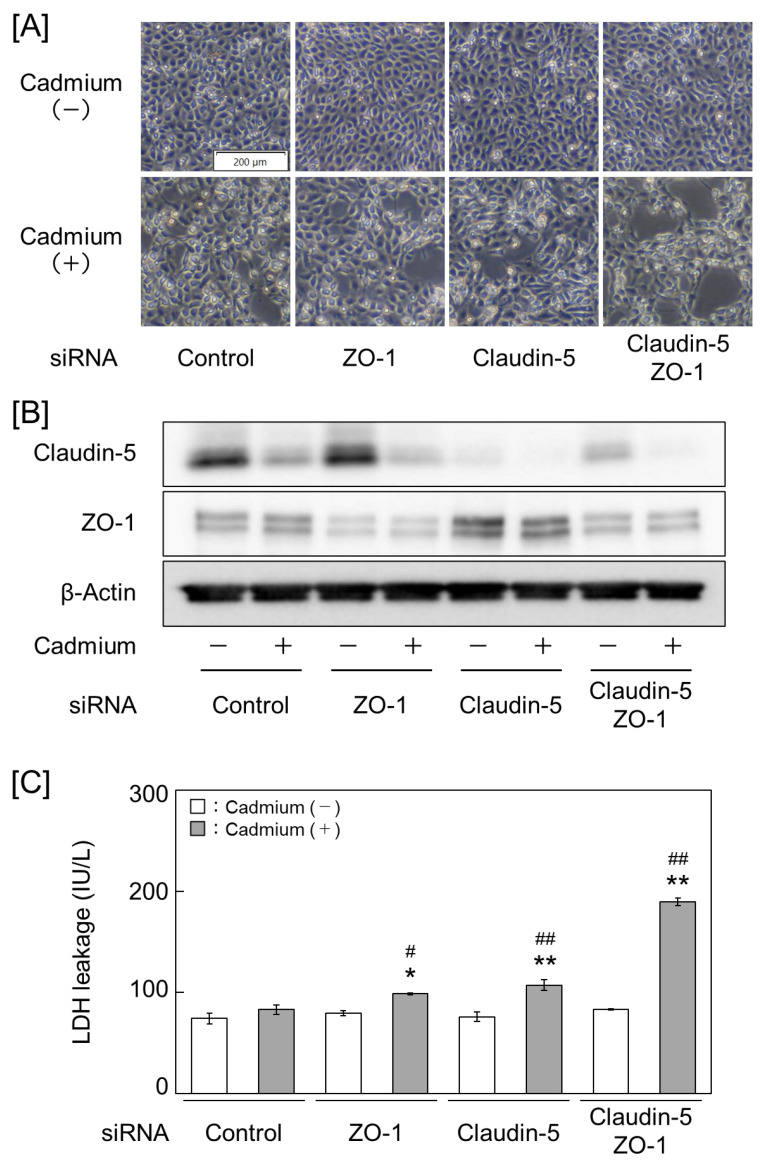
Cadmium toxicity is exacerbated in vascular endothelial cells that suppress the expression of claudin-5 and ZO-1. (**A**) Cell morphology. (**B**) Claudin-5 and ZO-1 protein expression levels. (**C**) Lactate dehydrogenase (LDH) leakage from cells. Vascular endothelial cells were treated with 2 μM cadmium for 24 h after transfection with a control, ZO-1, or claudin-5 small interfering RNA (siRNA). Values are represented as the mean ± S.E. of four samples. * *p* < 0.05 and ** *p* < 0.01 vs. without cadmium; ^#^ *p* < 0.05 and ^##^ *p* < 0.01 vs. corresponding control siRNA by Tukey’s test.

**Figure 5:**
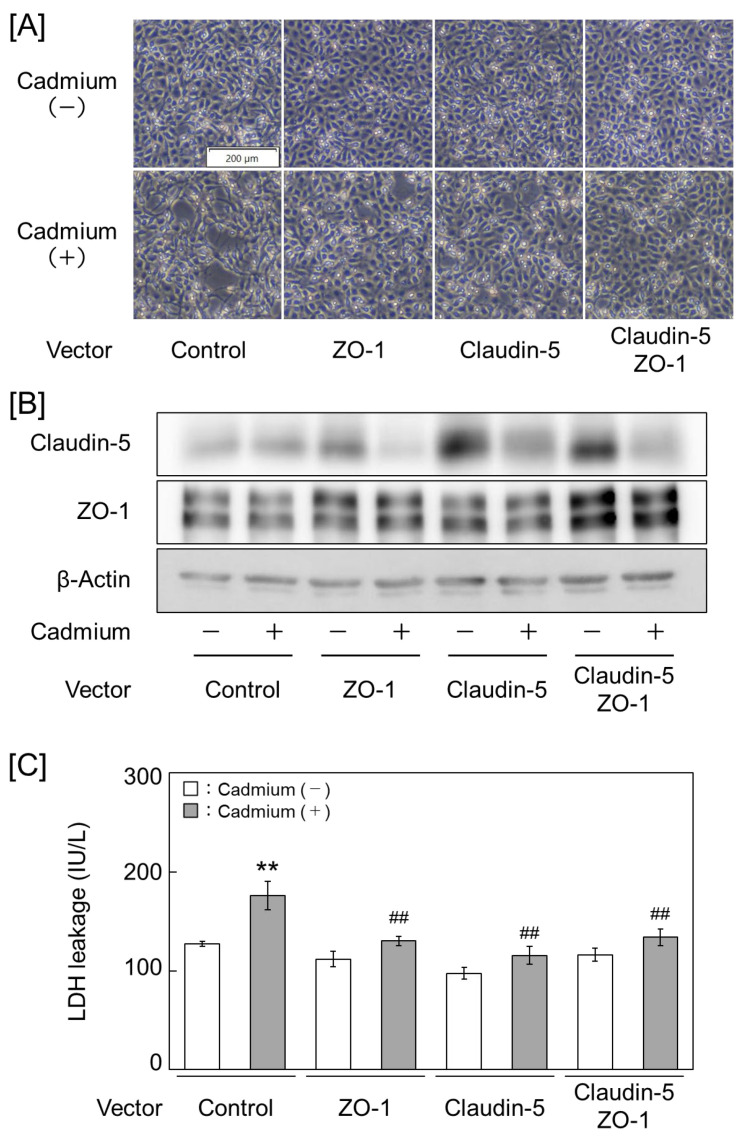
Cadmium toxicity is reduced in vascular endothelial cells overexpressing claudin-5 and ZO-1. (**A**) Cell morphology. (**B**) Claudin-5 and ZO-1 protein expression levels. (**C**) LDH leakage from cells. Vascular endothelial cells were treated with 2 μM cadmium for 24 h after transfection with the control, ZO-1, claudin-5, or claudin-5 and ZO-1 vector. Values are represented as the mean ± S.E. of four samples. ** *p* < 0.01 vs. without cadmium; ^##^ *p* < 0.01 vs. corresponding control vector by Tukey’s test.

As the expression levels of claudin-5 and ZO-1 were significantly reduced by cadmium, we examined whether these expression levels affect cell detachment using vascular endothelial cells. Cadmium-induced detachment was increased in the groups with inhibited claudin-5 and ZO-1 expression, along with enhanced lactate dehydrogenase (LDH) leakage from cells. Similarly, enhanced detachment and LDH leakage were observed in the groups with a simultaneous suppression of claudin-5 and ZO-1 (Figure 4 and Supplemental Figures S2B and S3C). In contrast, the groups with high claudin-5 and ZO-1 expression showed reduced cadmium-induced detachment and LDH leakage. Moreover, a simultaneous high expression of claudin-5 and ZO-1 significantly reduced cell detachment and LDH leakage in the cells (Figure 5 and Supplemental Figures S2C and S3D). These results suggest the tight junction components claudin-5 and ZO-1 as the key molecules involved in cadmium toxicity in vascular endothelial cells.

### 2.3. Cadmium-Activated CRE-Binding Protein (CREB) Inhibits Claudin-5 Expression and Promotes Endothelial Detachment

**Figure 6:**
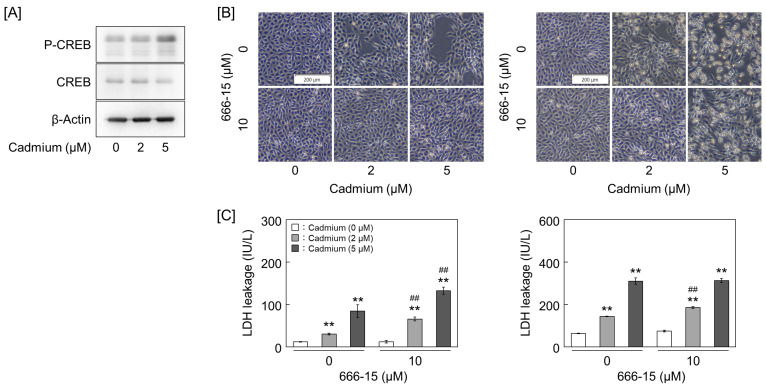
Effect of CRE-binding protein (CREB) inhibitor on cadmium toxicity of vascular endothelial cells. (**A**) CREB activation by cadmium treatment. Vascular endothelial cells were treated with 2 and 5 μM cadmium for 8 h. (**B**) Morphology of vascular endothelial cells. (**C**) LDH leakage from cells. Vascular endothelial cells were pretreated with 10 μM CREB inhibitor 666-15 for 2 h and then treated with 2 and 5 μM cadmium for 24 h (left panels) or 48 h (right panels). Values are represented as the mean ± S.E. of four samples. ** *p* < 0.01 vs. without cadmium; ^##^ *p* < 0.01 vs. without inhibitor by Tukey’s test.

Next, the mechanisms related to the inhibition of claudin-5 and ZO-1 expression and the protection of vascular endothelial cells from cadmium were investigated. CREB was activated under cadmium treatment (Figure 6A and Supplemental Figure S3E). Cadmium-induced cell detachment was significantly inhibited by the CREB inhibitor, 666-15 (Figure 6B and Supplemental Figure S2D). However, LDH leakage from cells was not inhibited by the CREB inhibitor (Figure 6C).

**Figure 7:**
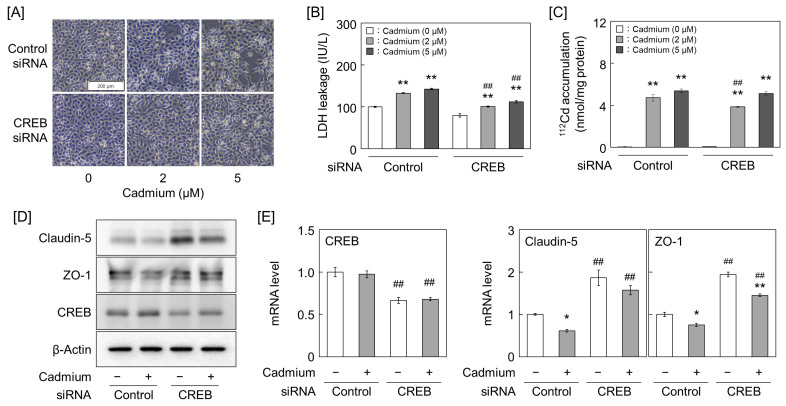
Effect of CREB knockdown on cadmium toxicity of vascular endothelial cells. (**A**) Morphology of vascular endothelial cells. (**B**) LDH leakage from cells. (**C**) Cadmium accumulation in cells. Vascular endothelial cells were transfected with the control or CREB siRNA for 24 h and then treated with 2 and 5 μM cadmium for 24 h. Values are represented as the mean ± S.E. of four samples. ** *p* < 0.01 vs. corresponding without cadmium; ^##^ *p* < 0.01 vs. corresponding control siRNA by Tukey’s test. (**D**,**E**) Protein and mRNA expression levels of claudin-5, ZO-1, and CREB in vascular endothelial cells. Vascular endothelial cells were transfected with the control or CREB siRNA for 24 h and then treated with 2 μM cadmium for (**E**) 12 h or (**D**) 24 h. Values are represented as the mean ± S.E. of triplicates. * *p* < 0.05 and ** *p* < 0.01 vs. corresponding without cadmium; ^##^ *p* < 0.01 vs. corresponding control siRNA by Tukey’s test.

Further tests using CREB-repressed cells showed that cadmium-induced cell detachment was reduced in the CREB-knockdown group, similar to that in the CREB inhibitor pretreatment group (Figure 7A and Supplemental Figure S2E). The CREB-knockdown group showed significantly low cadmium-induced LDH leakage (Figure 7B), suggesting that the increased LDH leakage in cells pretreated with the CREB inhibitor 666-15 (Figure 6C) is a non-specific effect unrelated to the suppression of cell detachment. We also measured cadmium accumulation in the cells and found that the CREB-knockdown group did not accumulate less cadmium than the control group at any of the tested cadmium concentrations (Figure 7C). The claudin-5 protein and mRNA levels were significantly increased by the CREB knockdown. However, the ZO-1 protein expression changes did not reflect the mRNA expression changes induced by the CREB knockdown. The CREB knockdown partially and significantly suppressed the cadmium-induced decrease in claudin-5 protein and mRNA expression, respectively. Additionally, the decrease in ZO-1 protein expression was suppressed by the CREB knockdown (Figure 7D,E and Supplemental Figure S3F).

## 3. Discussion

Vascular endothelial cells are the key targets of cadmium-induced toxicity [15]. We previously reported that cadmium induces pore-like desorption injury in the monolayer structure of vascular endothelial cells [8]. This study aimed to elucidate the underlying molecular mechanisms. We found that (1) tight-junction-related proteins of endothelial cells exhibited varying mRNA and protein levels in response to metal exposure, (2) the claudin-5 and ZO-1 levels were downregulated by cadmium, (3) decreased expression levels of claudin-5 and ZO-1 exacerbated cadmium-induced desorption, whereas increased expression levels of claudin-5 and ZO-1 exhibited reverse effects, (4) cadmium activated CREB in vascular endothelial cells, and the cadmium-induced toxicity of CREB was reduced under CREB inhibition, (5) CREB inhibition had no significant effect on cadmium accumulation in endothelial cells, and (6) CREB inhibition increased claudin-5 protein expression. Claudin-5 and ZO-1 are important factors in cadmium-induced endothelial cell detachment.

Cadmium weakens the tight junctions, increasing the permeability at the blood–brain and blood–testis barriers [16,17,18]. Among these studies, a case has been reported in which cadmium suppressed the expression of ZO-1 in rat brain vascular endothelial cells [19]. However, no study has reported the effect of cadmium on claudin-5 expression in vascular endothelial cells. This study is important in considering how tight junctions are weakened. The four heavy metals examined in this study showed different effects on the expression of tight junction component molecules in vascular endothelial cells. Only cadmium suppressed the expression of claudin-5, an intercellular junction molecule, and ZO-1, a lining protein. This may explain why detachment toxicity is cadmium-specific. We also reported that zinc pretreatment protects vascular endothelial cells against cadmium toxicity [9]. In this case, the accumulation of cadmium in endothelial cells was also reduced, which may have affected the expression of ZIP8 and ZIP14 transporters involved in uptake. However, given that zinc itself has a low metallothionein induction capacity in vascular endothelial cells [20], it is possible that the induction of claudin-5 and claudin-12 by zinc also contributes to the protection against cadmium-induced endothelial cell toxicity. We recently revealed that manganese pretreatment reduces the endothelial cytotoxicity of cadmium and that expression changes in ZIP8, ZIP-14, and metallothionein are not involved in this protection [21].

Cadmium suppresses protein tyrosine phosphatase (PTP) function via reactive oxygen species (ROS) and leads to blood–brain barrier dysfunction in zebrafish [22]. Arsenic enhances permeability via the ROS–vascular endothelial growth factor (VEGF) pathway in bEnd3-immortalized mouse brain microvascular endothelial cells [23]. As the VEGF receptor is phosphorylated by PTP1B, heavy metals reduce the activity of phosphatases, starting with the production of ROS, leading to an increased phosphorylation of downstream signals [24]. CREB is activated by cadmium [25], and PTEN, a phosphatase, inhibits CREB activation [26], suggesting CREB activation via PTEN inhibition [27] by cadmium as a possible mechanism for this phenomenon. In a study using bEnd.3, CREB activation and claudin-5 expression were positively correlated [28], whereas the present results using bovine aortic endothelial cells showed a negative correlation between CREB activation and claudin-5 expression. While the reason for the opposite effect of CREB is not known, it has been shown through JASPAR that the bovine claudin-5 promoter region, as in mouse, contains two regions like the predicted CRE binding sequence (5′-TGACGTCA-3′) at -1519 TGAAGTCT -1526 and -576 TGAGGTCT -569, suggesting that differences in vascular regions or animal species affect the regulation of claudin-5 expression.

Decreased claudin-5 and ZO-1 expression levels in vascular endothelial cells indicate weakened tight junctions and an enhanced transport of ions and macromolecules via the paracellular pathway [14]. Furthermore, weakened adhesion between endothelial cells facilitates the trans-endothelial migration of monocytes. The expression levels of claudin-5, ZO-1, and occludin are decreased in the cerebral microvessels of mice fed a high-fat diet [29], indicating that the combined effects of diet and cadmium exposure accelerate the development and progression of atherosclerosis. Injury and detachment of endothelial cells also lead to ischemic heart disease owing to increased thrombogenicity in subendothelial tissues. Overall, this study showed that claudin-5 and ZO-1 are the key molecules involved in cadmium-induced endothelial cell detachment and that an increase in their expression levels prevents cadmium-induced endothelial cell detachment.

## 4. Materials and Methods

### 4.1. Materials

Bovine aortic endothelial and smooth muscle cells were purchased from Cell Applications (San Diego, CA, USA). Tissue culture dishes and plates were obtained from AGC Techno Glass (Shizuoka, Japan). Dulbecco’s modified Eagle’s medium (DMEM) and Ca^2+^- and Mg^2+^-free phosphate-buffered saline were obtained from Nissui Pharmaceutical (Tokyo, Japan). Fetal bovine serum (FBS) was purchased from Biosera (Kansas City, MO, USA). ISOGEN II, Gene Ace SYBR quantitative polymerase chain reaction (qPCR) Mix α, gene-specific small interfering RNAs (siRNAs), and universal negative control siRNA were obtained from Nippon Gene (Toyama, Japan). ReverTra Ace qPCR RT Master Mix was purchased from TOYOBO (Osaka, Japan). The pBI-CMV1 vector, PrimeSTAR Max DNA Polymerase, and PrimeSTAR GXL DNA polymerase were purchased from Takara Bio (Shiga, Japan). Claudin-5 (NM_001130861) human tagged ORF clone was purchased from OriGene (Rockville, MD, USA). ZO-1 (NM_003257.5) human tagged ORF clone was obtained from GenScript (Piscataway, NJ, USA). NEBuilder HiFi DNA Assembly Master Mix was obtained from _New England Biolabs (Ipswich, MA, USA). Amersham Hybond P PVDF 0.2 was purchased from Cytiva (Marlborough, MA, USA). A Pierce BCA protein assay kit, lipofectamine RNAiMAX, and lipofectamine LTX were purchased from Thermo Fisher Scientific (Waltham, MA, USA). A CytoTox 96 Non-Radioactive Cytotoxicity Assay Kit was purchased from Promega (Madison, WI, USA). Anti-claudin-5 (ab131259) and anti-ZO-3 (ab204231) antibodies were purchased from Abcam (Cambridge, UK). Anti-claudin-12 antibody (#18801) was obtained from IBL (Gunma, Japan). Anti-ZO-1 (21773-1-AP) and anti-occludin (13409-1-AP) antibodies were purchased from Proteintech (Rosemont, IL, USA). Anti-ZO-2 (#2847), anti-CREB (#9197), anti-phospho-CREB (#9198), horseradish peroxidase-conjugated anti-rabbit IgG (#7074), and anti-mouse IgG (#7076) antibodies were purchased from Cell Signaling Technology (Danvers, MA, USA). β-Actin antibody (010-27841) and zinc sulfate heptahydrate were obtained from Fujifilm Wako Pure Chemical Industries (Osaka, Japan). Sodium (meta)arsenite and CREB inhibitor 666-15 were purchased from Merck (Darmstadt, Germany). Cadmium chloride, manganese(II) chloride tetrahydrate, Chemi-Lumi One Super, and other reagents were purchased from Nacalai Tesque (Kyoto, Japan).

### 4.2. Cell Culture and Treatment

Bovine aortic endothelial cells were cultured separately in a humidified atmosphere containing 5% CO_2_ at 37 °C in DMEM supplemented with 10% FBS until confluence. The cells were transferred to 35 mm dishes or 6-/24-well plates with DMEM supplemented with 10% FBS and cultured until confluence. Subsequently, the medium was discarded, and the cells were washed twice with serum-free DMEM, followed by treatment with cadmium, arsenite, and manganese (1, 2, 3, 4, and 5 µM each) or zinc (10, 20, 30, 40, and 50 µM each) for 4, 8, 12, 24, and 48 h. Detailed experimental conditions are described in the figure legends.

### 4.3. siRNA Transfection

siRNA transfection was performed using lipofectamine RNAiMAX as previously described [30], with slight modifications. Briefly, annealed siRNA duplexes and lipofectamine RNAiMAX were dissolved in Opti-MEM in separate tubes, left for 5 min, mixed, and left again for 20 min. Vascular endothelial cells were cultured until subconfluency in DMEM with 10% FBS and incubated at 37 °C in a fresh DMEM supplemented with 10% FBS in the presence of the siRNA/lipofectamine RNAiMAX mixture. The final concentrations of siRNA and lipofectamine RNAiMAX were 18 nM and 0.09%, respectively. After 24 h, the medium was changed to fresh DMEM, and the cells were treated with cadmium for 12 or 24 h. All siRNA sequences used are listed in Table 1.

### 4.4. Construction of a Bidirectional Expression Plasmid Vector

Claudin-5 expression plasmid and ZO-1 and claudin-5 and ZO-1 co-expression plasmids were constructed using the pBI-CMV bidirectional expression vector.

#### 4.4.1. Claudin-5 Expression Plasmid

PCR was performed using the PrimeSTAR Max DNA polymerase and claudin-5 human tagged ORF clone as a template with the following primers: forward primer 5′-TTTGAATTCATGACCCGCGCACGGGAT-3′ and reverse primer 5′-TTTTTCTAGATTAGACGTAGTTCTTCTTGTCGTAGTCG-3′. The resulting amplicons and pBI-CMV1 were digested by *Eco*RI and *Xba*I and assembled.

#### 4.4.2. ZO-1 Expression and Claudin-5 and ZO-1 Co-Expression Plasmids

PCR was performed using the PrimeSTAR GXL DNA polymerase and ZO-1 human tagged ORF clone as a template with the following primers: forward primer 5′-GAACCGTCAGATCCGCTAGGATGCGGCGCCTGGAGGGG-3′ and reverse primer 5′-CTCTGGAGATATCGTCGACATTAAAAGTGGTCAATAAGGACAGAAACACAGTTTGCTCCAAC-3′. The resulting amplicons were assembled using the pBI-CMV1 or claudin-5 expression plasmid, which were digested by *Bam*HI and *Hind*III using the NEBuilder HiFi DNA Assembly Master Mix.

### 4.5. Plasmid Vector Transfection

Cell transfection of plasmid DNA was performed using lipofectamine LTX, as previously described [31], with slight modifications. Plasmid DNA and PLUS reagent in Opti-MEM and lipofectamine LTX in Opti-MEM were prepared in separate tubes and mixed together. The mixture was left for 5 min at room temperature. Vascular endothelial cells were cultured until subconfluency in DMEM supplemented with 10% FBS and incubated at 37 °C in fresh DMEM supplemented with 10% FBS in the presence of the plasmid DNA/lipofectamine LTX mixture. The final concentrations of the plasmid DNA, PLUS reagent, and lipofectamine LTX were 0.5 µg/mL, 0.05%, and 0.10%, respectively. After 24 h, the medium was changed to fresh DMEM, and the cells were treated with cadmium for 24 h.

### 4.6. Western Blotting

Claudin-5, claudin-12, occludin, ZO-1, ZO-2, ZO-3, (phospho-)CREB, and β-actin proteins were separated via sodium dodecyl sulfate–polyacrylamide gel electrophoresis, and Western blotting was performed as previously described [24]. Immunoreactive bands were visualized using the Chemi-Lumi One Super Western blot detection reagent and scanned using an Amersham Imager 600 (GE Healthcare).

### 4.7. Total RNA Extraction and Quantitative Reverse Transcription (qRT)-PCR

mRNA expression was analyzed using qRT-PCR on a CFX Connect Real-Time PCR Detection System (BioRad, Hercules, CA, USA) after total RNA extraction, as previously described [24]. Levels of claudin-5 and -12, occludin, ZO (TJP)-1, -2, and -3, CREB (CREB1), and glyceraldehyde 3-phosphate dehydrogenase (GAPDH) were quantified using the relative standard curve method. The fold-change in the intensity of the target gene was normalized to that of GAPDH. All primer sequences are listed in Table 2.

### 4.8. Cytotoxicity Assay

Next, a cytotoxicity assay was performed. Briefly, vascular endothelial cells cultured under each condition in a 6- or 12-well culture plate were treated with cadmium for 24 or 48 h. After incubation, a portion of the treated medium was collected, and LDH activity (marker for cell death) was measured using a CytoTox 96 Non-Radioactive Cytotoxicity Assay kit.

### 4.9. Intracellular Accumulation of Cadmium

Control or CREB siRNA-transfected vascular endothelial cells cultured in a 6-well plate were treated with cadmium for 24 h. After incubation, the cells were lysed with 100 μL of 50 mM Tris-HCl buffer (pH 6.8) containing 2% sodium dodecyl sulfate and 10% glycerol. The cells were then lysed by incubating at 95 °C for 10 min, and 60 µL of the lysate was incubated at 130 °C for 48 h in 9.6 M nitric acid containing 7.4% hydrogen peroxide. The samples were dried and dissolved in 4 mL of 0.1 M nitric acid. The content of cadmium atoms was analyzed using inductively coupled plasma mass spectrometry (ICP-MS) (Nexion 300S, PerkinElmer, Waltham, MA USA). The conditions of ICP-MS were optimized for a plasma output of 1600 W, plasma gas flow rate of 18.0 L/min, and nebulizer gas flow rate of 0.96 L/min. Another portion of the cell lysate was analyzed for the protein concentration using a Pierce BCA protein assay kit, and the cadmium content was expressed as nmol/mg protein.

### 4.10. Statistical Analyses

Data are represented as the mean ± standard deviation of three or four samples analyzed using Student’s *t*, Dunnett’s, or Tukey’s tests, as required. Statistical significance was set at *p* < 0.05.

## 5. Conclusions

This study revealed that cadmium causes vascular endothelial cell detachment by inhibiting the expression of the tight junction proteins claudin-5 and ZO-1, with CRE-binding protein activation playing a key role in this process.

## Figures and Tables

**Table 1:** The sequence of bovine-specific siRNAs.

Target	Sense (5′-3′)	Antisense (5′-3′)
Claudin-5 (CLDN5)	ccggcgacuaugacaagaadTdT	uucuugucauagucgccggdTdT
ZO-1 (TJP1)	uuuaacuugcccuuagaccdTdT	ggucuaagggcaaguuaaadTdT
CREB (CREB1)	ucauuugcuggcuuucagcdTdT	gcugaaagccagcaaaugadTdT

**Table 2:** The sequence of bovine mRNA specific primers.

Target	Sense (5′-3′)	Antisense (5′-3′)
CLDN5	GTCTCAGAAGTACGAGCTGGG	GTACTTCACCGGGAAGCTGAA
CLDN12	CAATAGTGCGGGCTGCCATC	AAAGACTGGCTCGAACTTCCTGT
OCLN	GTGCATCGCCATTTTCGCCT	AACCGTAGCCATAGCCGTAGC
TJP1	CCTCTTGAGCCTTGAACTTTGAC	ACACTTTAGGGCACAGCATCG
TJP2	CAGCCCAGAGAGACACCAC	AGCAAAACCCTCTCGTAGGC
TJP3	GGATTCTAGGACCGCGTCAG	GCCTGAACCCCGGATACAAA
CREB1	ACATACCAGATTCGCACAGC	TTCTTTCTTCTTTCTACGACACTC
GAPDH	GGATCTGCTCCTGGAAGATG	CAATGACCCCTTCATTGACCTTC

## Data Availability

The original contribution presented in the study are included in the article/Supplementary Materials, further inquiries can be directed to the corresponding authors.

## Supplementary Materials

The following supporting information can be downloaded at: https://www.mdpi.com/article/10.3390/ijms252011035/s1.
