# Supplementary material for: Cadmium Induces Vascular Endothelial Cell Detachment by Downregulating Claudin-5 and ZO-1 Levels
Source: Int J Mol Sci. 2024 Oct 14;25(20):11035. doi: 10.3390/ijms252011035 (PMC11507107; doi:10.3390/ijms252011035)
Supplement: Supplementary file 1 [file ijms-25-11035-s001.zip › supplementary figures 1-3.pdf]

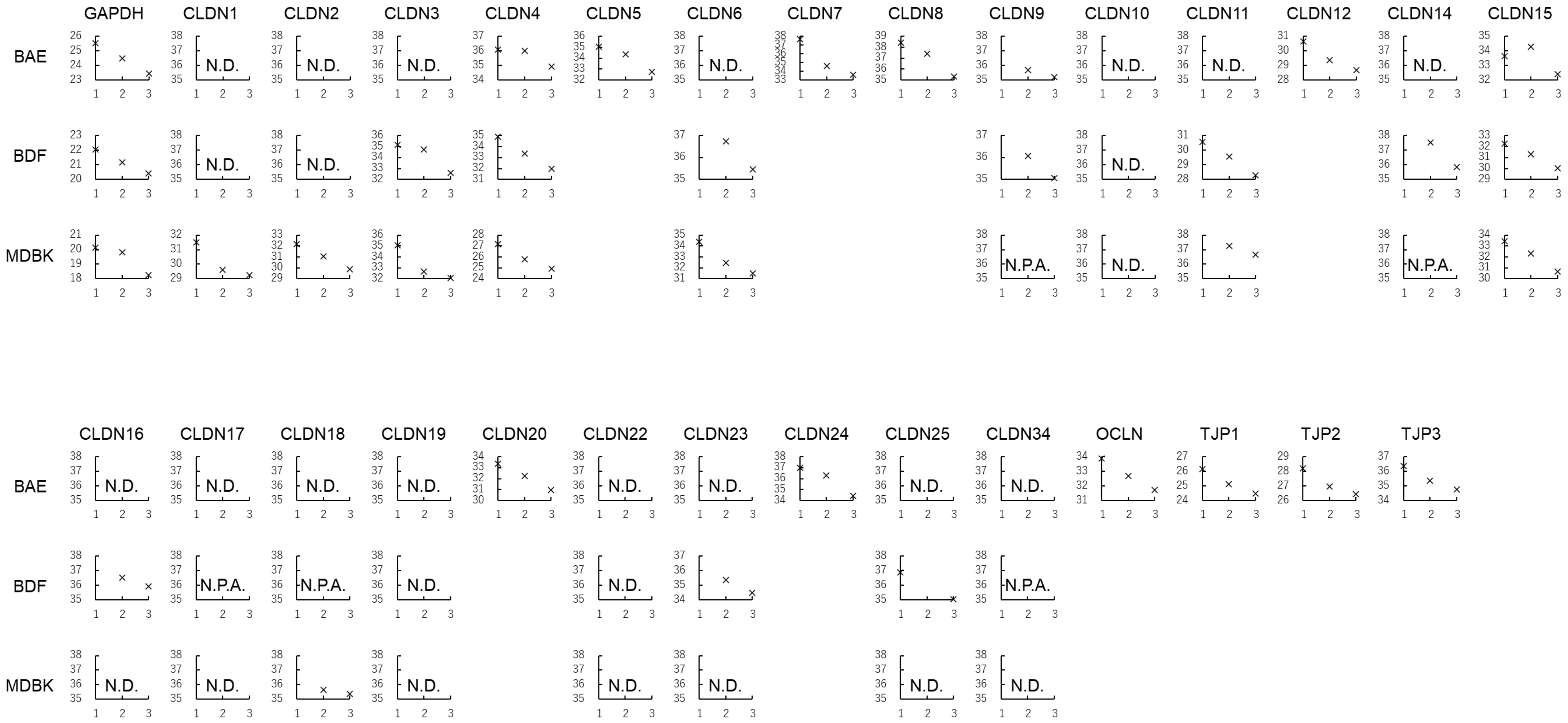

Supplemental Figure 1. Verification of mRNA expression of tight junction-related molecules.

The mRNA expression of all genes was first confirmed using bovine aortic endothelial cells (BAE), and additional analyses were performed using bovine dermal fibroblasts (BDF) and Madin-Darby bovine kidney cells (MDBK) for genes that were not detected in BAE. The x-axis represents the amount of template used for qRT-PCR (2^x ng/well) and the y-axis represents cycle threshold (Ct). N.D. means not detected and N.P.A. means not properly amplified.

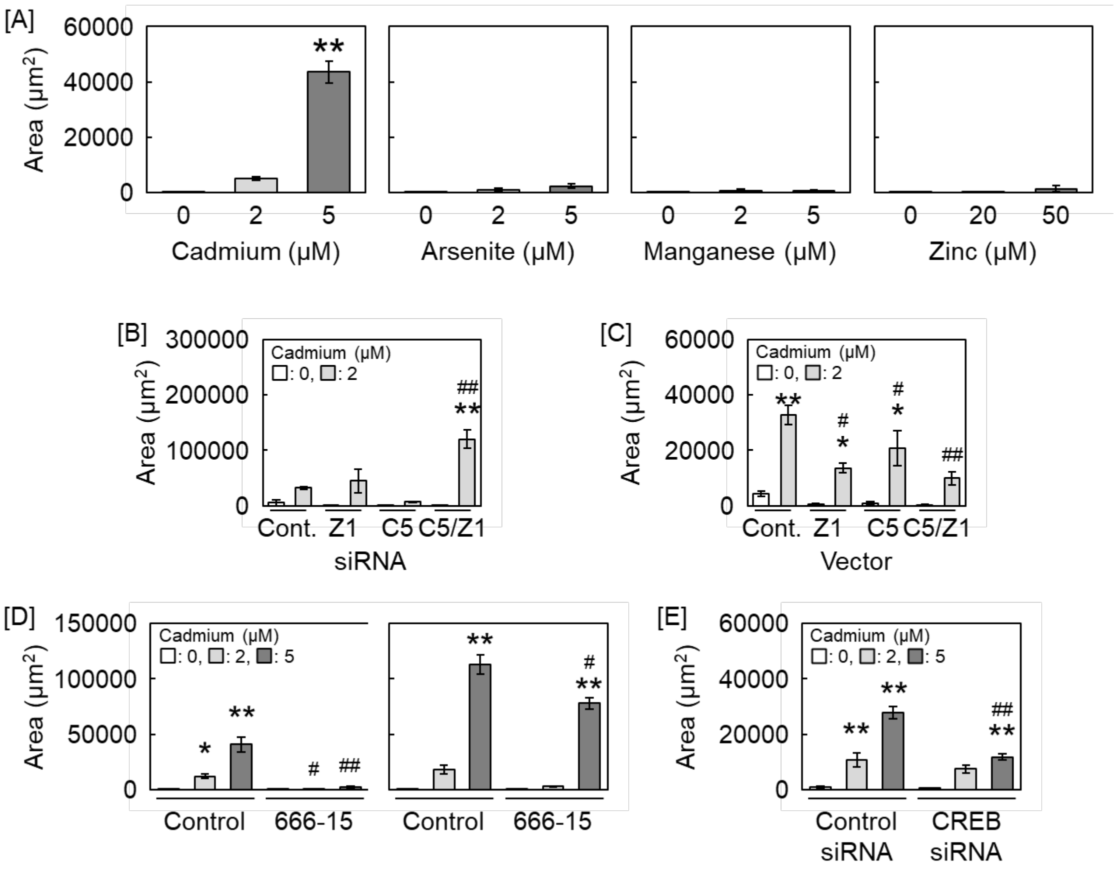

Supplemental Figure 2. Measurement of detachment area of vascular endothelial cells

The detached area of vascular endothelial cells in the experiments of Figure [A] 1A, [B] 4A, [C] 5A, [D] 6B and [E] 7A were analyzed using cellSens software (Olympus, Japan). The area of cell detachment was measured from photos of 872000 μm² per location. Values are represented as the mean ± standard error (S.E.) of triplicates. **p* < 0.05 and ***p* < 0.01 vs. without cadmium; #*p* < 0.05 and ##*p* < 0.01 vs. corresponding without inhibitor or control siRNA by Tukey's test.

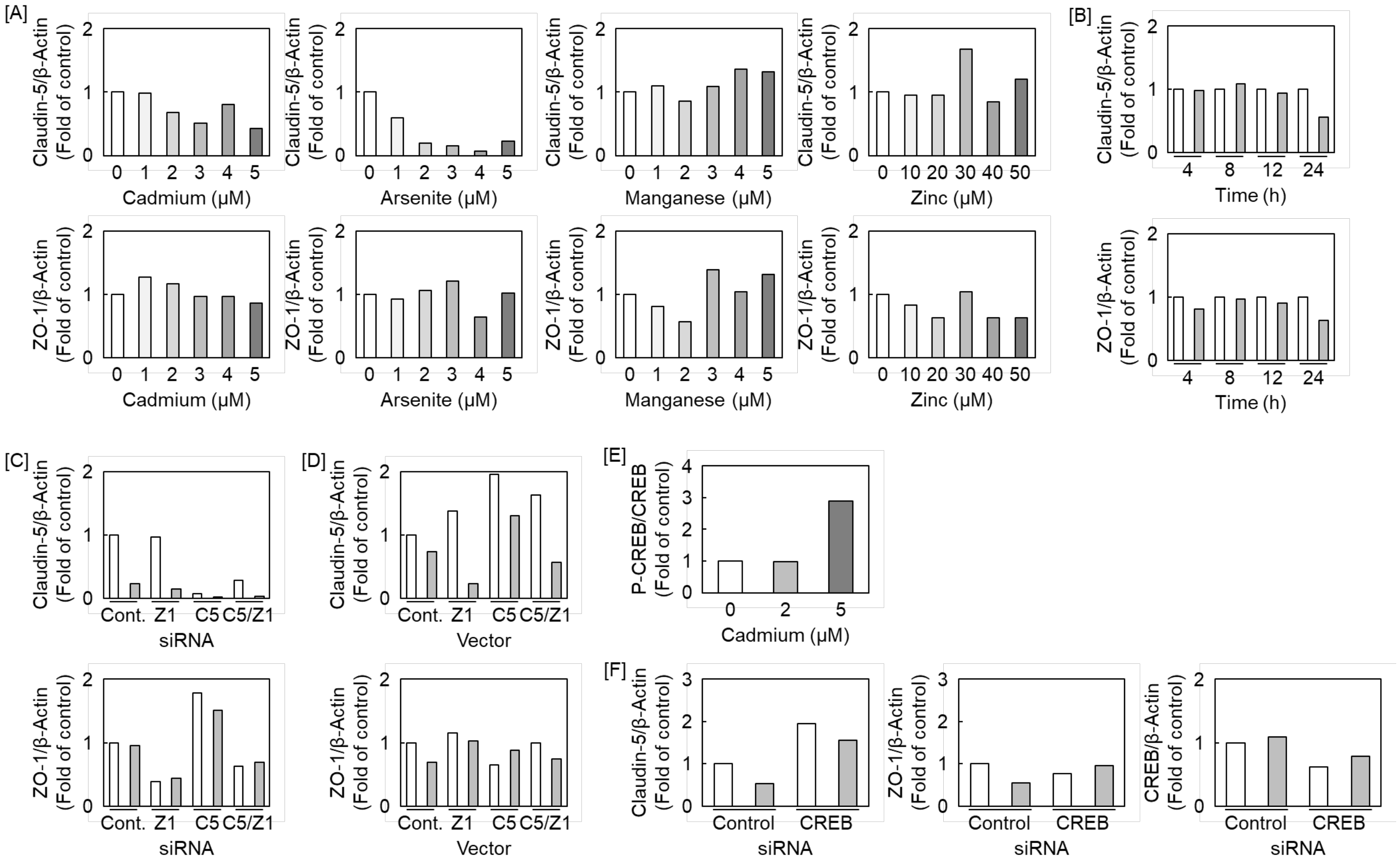

Supplemental Figure 3. Densitometry analysis of Western blot bands

Densitometry analysis was performed to the Western blot bands in the Figure [A] 2, [B] 3B, [C] 4B, [D] 5B, [E] 6A, and [F] 7D using ImageJ. Each band intensity was corrected for the corresponding β-actin or CREB band intensity.
